# The association between therapeutic plasma exchange and the risk of mortality among patients critically ill with COVID-19: a meta-analysis

**DOI:** 10.12688/f1000research.74972.1

**Published:** 2021-12-14

**Authors:** Shinta Oktya Wardhani, Jonny Karunia Fajar, Gatot Soegiarto, Laksmi Wulandari, Helnida Anggun Maliga, Muhammad Ilmawan, Risna Merysa, Arlentina Bentivolia Simamora, Qurrata Aini, Komang Noviantari, Ayu Widya Lestari, Maria Yoheni Harnila, Imam Syafi'i, Jane Andrea Christiano Djianzonie, Nenci Siagian, Sri Nining, Risyda Zakiyah Hanim, Wahyuni Wahyuni, Fitri Aulia, Juliansyah Juliansyah, Reflin Mahmud, Fredo Tamara, Aditya Indra Mahendra, Amanda Cininta Wowor, Fikri Baladraf, Ponda Hernest Hadinata, Adhityari Ikkeputri, Hana Nadya, Dessy Aprilia Kartini, Milda Husnah, Firzan Nainu, Harapan Harapan

**Affiliations:** 1 Division of Hematology and Oncology, Department of Internal Medicine, Faculty of Medicine, Universitas Brawijaya, Malang, East Java, 65145, Indonesia; 2 Brawijaya Internal Medicine Research Center, Department of Internal Medicine, Faculty of Medicine, Universitas Brawijaya, Malang, East Java, 65145, Indonesia; 3 Division of Allergy & Immunology, Department of Internal Medicine, Faculty of Medicine, Universitas Airlangga, Surabaya, East Java, 60286, Indonesia; 4 Department of Pulmonology and Respiratory Medicine, Faculty of Medicine, Universitas Airlangga, Surabaya, East Java, 60286, Indonesia; 5 Faculty of Medicine, Universitas Brawijaya, Malang, East Java, 65145, Indonesia; 6 Faculty of Nursing, Universitas Indonesia, Jakarta, 10430, Indonesia; 7 Faculty of Nursing, Universitas Syiah Kuala, Banda Aceh, Aceh, 23111, Indonesia; 8 Faculty of Pharmacy, Universitas Indonesia, Jakarta, 10430, Indonesia; 9 Department of Internal Medicine, Faculty of Medicine, Universitas Indonesia, Jakarta, 10430, Indonesia; 10 Department of Internal Medicine, Faculty of Medicine, Universitas Airlangga, Surabaya, East Java, 60286, Indonesia; 11 Faculty of Public Health, Universitas Indonesia, Jakarta, 10430, Indonesia; 12 Medical Research Unit, School of Medicine, Universitas Syiah Kuala, Banda Aceh, Aceh, 23111, Indonesia; 13 Faculty of Mathematics and Natural Sciences, Universitas Syiah Kuala, Banda Aceh, Aceh, 23111, Indonesia; 14 Faculty of Pharmacy, Hasanuddin University, Tamalanrea, Makassar, 90245, Indonesia; 15 Tropical Disease Centre, School of Medicine, Universitas Syiah Kuala, Banda Aceh, Aceh, 23111, Indonesia; 16 Department of Microbiology, School of Medicine, Universitas Syiah Kuala, Banda Aceh, Aceh, 23111, Indonesia

**Keywords:** COVID-19; therapeutic plasma exchange; cytokine storm; treatment

## Abstract

**Background:** Cytokine storm has been widely known to contribute to the development of the critical condition in patients with coronavirus disease 2019 (COVID-19), and studies had been conducted to assess the potential aspect of cytokine storm elimination by performing therapeutic plasma exchange (TPE). However, contradictory findings were observed. The objective of this study was to assess the association between TPE and the reduction of mortality of critically ill COVID-19 patients.

**Methods: **A meta-analysis was conducted by collecting data from PubMed, Scopus, and Web of Science. Data on the mortality rate of critically ill COVID-19 patients treated with TPE plus standard of care and that of patients treated with standard of care alone were analyzed using a Z test.

**Results:** We included a total of four papers assessing the association between TPE and the risk of mortality among critically ill COVID-19 patients. Our findings suggested that critically ill COVID-19 patients treated with TPE had lower risk of mortality compared to those without TPE treatment.

**Conclusion:** Our study has identified the potential benefits of TPE in reducing the risk of mortality among critically ill COVID-19 patients.

## Introduction

Since first reported in December 2019, ^1^ coronavirus disease 2019 (COVID-19) has become an unresolved global pandemic. The challenge of the pandemic management at the present time might be due to the fact that a number of mutations have occurred making the virus more transmissible and causing critical illness. ^2^ The World Health Organization (WHO) has established a living guideline on drugs for the management of COVID-19 ^3^ and updated it periodically. However, the treatment of critically ill COVID-19 patients remains a serious issue. ^4^ Patients critically ill with COVID-19 have been widely reported to have a poor prognosis, and theory reveals that cytokine storm might underlie this mechanism. In a cytokine storm excessive accumulation of pro-inflammatory cytokines might be responsible for the poor prognosis of COVID-19 patients. No study has found an effective treatment for the management of a cytokine storm in patients critically ill with COVID-19. Therefore, an investigation into the treatment that acts to remove these pro-inflammatory cytokines, for example, using therapeutic plasma exchange (TPE) may be required.

Since first introduced in 1952, TPE has been shown to provide an excellent outcome in patients with multiple myeloma to control hyperviscosity. ^5^ Moreover, the implementation of this therapeutic treatment has also been reported in an *Escherichia coli* outbreak, ^6^ a Shigella infection, ^7^ infectious toxicosis, ^8^ and septic shock with multiple organ failure ^9^; and reduced risk of mortality was revealed. In the case of COVID-19, the US Food and Drug Administration (FDA) has posited that TPE may have a role as a rescue therapy in critically ill patients with COVID-19. ^10^ However, insufficient evidence has resulted in indecision in applying TPE for the management of critically ill COVID-19 patients. To date, TPE has been studied in Oman, ^11^ Turkey, ^12^ Pakistan, ^13^ and Saudi Arabia. ^14^ However, contradictory findings exist. Therefore, our study aimed to assess the potential of TPE in reducing mortality of critically ill COVID-19 patients using a meta-analysis approach. The findings might add new insight and clarify the true potency of TPE for treating patients critically ill with COVID-19.

## Methods

### Study design

From March to August 2021, a meta-analysis following the protocols of the Preferred Reporting Items for Systematic Reviews and Meta-analyses (PRISMA) ^15^ was conducted to evaluate the effectiveness of TPE in reducing the mortality rate of critical COVID-19 patients. The PRISMA checklist in our present study is presented as extended data in Figshare. ^16^ The major databases including PubMed, Scopus, and Web of Science were used to search for potential articles.

### Eligibility criteria

Inclusion and exclusion criteria were defined to assess relevant articles. The inclusion criteria of the study were (1) observational or randomized controlled trial studies, (2) having adequate information to calculate the potential association and effect estimates, and (3) applying a well-defined methodological approach to establish a COVID-19 diagnosis. All case reports, case series, letters to the editor, reviews, and commentaries, as well as studies with pre-post test comparison, and poor-quality methodology assessed with the Newcastle-Ottawa scale (NOS) were excluded.

### Search strategy and data extraction

The source databases used in our study were PubMed, Scopus, and Web of Science. We restricted the searching period up to 28 July 2021, and the language was English only. The Medical Subject Headings were: (“COVID-19” or “SARS-CoV-2”) and (“plasma exchange” or “therapeutic plasma exchange” or “TPE”). The reference lists of all potential related articles were also assessed to retrieve additional relevant articles. Data extraction was performed for all included papers, including: (1) name of the first author; (2) year of publication; (3) country of origin; (4) sample size of cases and controls, (5) TPE, and (6) mortality rate between groups.

### Assessment of the methodology quality

All included articles were assessed for their quality using NOS for observational studies ^17^ and the Jada-modified scale for RCTs. ^18^ The article quality was interpreted as low, moderate, and high. Low quality articles were excluded from our study. The assessment was performed by two independent authors (MI, HAM), and when a discrepancy was observed an assessment by a senior researcher (JKF) was conducted.

### Outcome measure

The main outcome of the study was all causes of mortality among critical COVID-19 patients treated with and without TPE. The diagnosis of COVID-19 was established by using RT-PCR of SARS-CoV-2 RNA from nasal or oropharyngeal swab samples, and critical COVID-19 patients were defined by following the guideline (requires life sustaining treatment, acute respiratory distress syndrome, sepsis, or septic shock). ^3^^,^^19^

### Statistical analysis

The calculation of potential publication bias, heterogeneity among studies, and the association between the use of TPE and the risk of mortality among patients with COVID-19 were assessed using an Egger test, a Q test, and a Z test; respectively. The Egger test with a p-value more than 0.05 indicated the presence of potential publication bias. Moreover, the heterogeneity among studies was considered when the p-value of a Q test indicated less than 0.10. The pooled association was calculated using a Z test, where the p-value of less than 0.05 indicates a significant association. The estimated effect was presented as an odds ratio with 95% confidence interval (OR 95% CI). The cumulative calculation was presented as a forest plot. An R package software ( R Studio version 4.1.1, MA, USA, (RRID:SCR_000432) was used to perform the analyses.

## Results

### Studies selection

**Figure 1.**
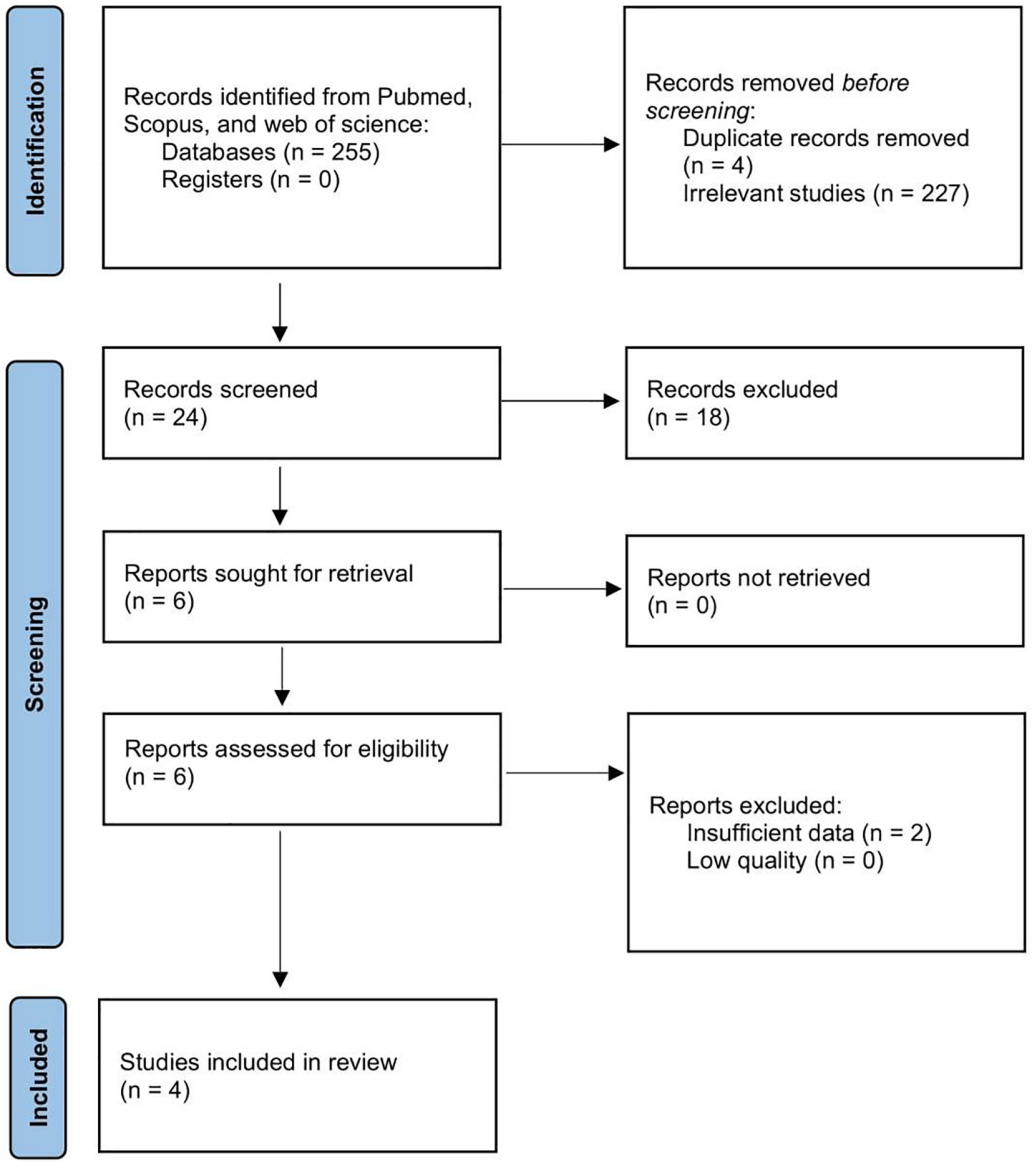
Preferred Reporting Items for Systematic Reviews and Meta-Analyses (PRISMA) flowchart of article selection in our meta-analysis.

We identified a total of 255 papers. Among them, four papers were excluded due to duplication and additional 227 papers due to irrelevant context. There were 24 papers in total included for full-text assessment. Then, 20 of the 24 papers were further excluded since 18 were reviews and case reports and two papers had insufficient data. Four papers were included in the final analysis. ^11^^–^^14^ The article selection PRISMA flowchart is presented in Figure 1 and the baseline characteristics are described in Table 1.

**Table 1.** Baseline characteristics of articles included in our study.

Study and year	Country	Study design	Quality	TPE		Control	
				Total	Mortality	Total	Mortality
Khamis *et al*., 2020 ^11^	Oman	Cohort Retrospective	High	11	1	20	9
Gucyetmez *et al*., 2020 ^12^	Turkey	Cohort Retrospective	Moderate	12	1	12	7
Kamran *et al*., 2020 ^13^	Pakistan	Cohort Retrospective	High	45	4	45	18
Faqihi *et al*., 2020 ^14^	Saudi Arabia	RCT	High	43	9	44	15

### TPE treatment and COVID-19 mortality rate

**Figure 2.**
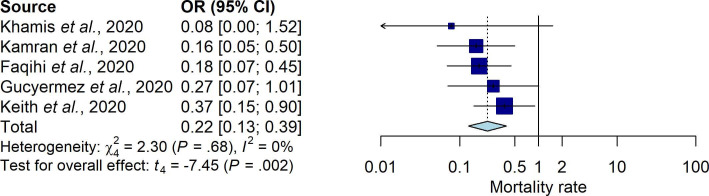
Forest plot of mortality rate between therapeutic plasma exchange vs control (OR: 0.2097; 95% CI: 0.0516, 0.852; p-value: 0.0382; pHet: 0.2065; pE: 02153).

A total of 111 COVID-19 patients treated with TPE and 121 COVID-19 patients without TPE, retrieved from three retrospective cohort studies and one RCT, were included in our analysis. Our results found that COVID-19 patients treated with TPE had reduced mortality rate compared to COVID-19 patients without TPE treatment (OR: 0.21; 95% CI: 0.05, 0.85) ( Figure 2).

### Heterogeneity and potency of bias across the studies

Our analysis revealed the absence of the evidence of heterogeneity. Therefore, we applied a fixed-effect model to assess the correlation. For the potency of bias assessment across the studies, our analysis using an Egger test found no publication bias.

## Discussion

Our study identified that TPE treatment in critically ill COVID-19 patients reduced the mortality rate. To date, our study is the first meta-analysis to report on the use of TPE for the management of COVID-19. In our analyses, we included four studies from Oman, ^11^ Turkey, ^12^ Pakistan, ^13^ and Saudi Arabia ^14^; and all reports revealed similar findings in which TPE treatment reduced mortality among patients with COVID-19. TPE has been applied and proved to reduce the risk of mortality in the management of several infectious diseases, such as *Escherichia coli* O157:H7-associated hemolytic uremic syndrome, ^6^^,^^20^ Shigella infection, ^7^ infectious toxicosis, ^8^ HIV infection, ^21^ peripheral HIV neuropathy, ^22^ Kaposi's sarcoma, ^21^ disseminated cryptococcosis, ^23^ and septic shock with multiple organ failure. ^9^ Moreover, in the case of the *Escherichia coli* O157 outbreak in 1996, TPE proved beneficial in the reduction of mortality. ^6^ Therefore, as suggested in our study, TPE might possess potential benefits in COVID-19 treatment.

The precise mechanism of how TPE benefits COVID-19 patients remains debatable. In critical COVID-19 patients, the excessive accumulation of cytokines may occur, and this can lead to a fatal outcome. Previous studies have revealed that the levels of pro-inflammatory cytokines/chemokines including interleukin-2 (IL-2), interleukin-6 (IL-6), granulocyte colony stimulating factor (GCSF), IFN-γ inducible protein 10, monocyte chemoattractant protein 1 (MCP-1), macrophage inflammatory protein 1A, tumor necrosis factor-α (TNF-α) were observed to be higher in patients critically ill with COVID-19 compared to those with mild-moderate disease. ^24^^,^^25^ TPE is a therapeutic procedure principally acting to remove (through double filtration) molecules of 60–140 nm in size. ^5^ The molecule size of pro-inflammatory cytokines/chemokines is 80–220 nm. ^26^ Therefore, the elimination of pro-inflammatory cytokines/chemokines, proven to affect those critically ill with COVID-19 might provide benefits to improve the prognosis of COVID-19 patients. Moreover, a previous study also reported that TPE played an important role in eliminating toxic substances by suppressing the cytokine release syndrome. ^27^ It was also suggested that TPE plays a crucial role in restoring normal substances that may be deficient in the plasma, ^5^ leading to stabilization and restoration of endothelial membranes. ^28^ Another possibility is when fresh frozen plasma was used in fluid replacement; TPE was associated with the improvement of coagulopathy in COVID-19 patients. ^29^ Previous evidence suggests that TPE might play an important role in maintaining the balance between anti and pro-inflammatory cytokines in the plasma, and might rectify the prognosis in patients with COVID-19, as reported in our study.

To the best of our knowledge, our study is the first meta-analysis reporting the benefit of TPE in reducing the mortality rate of critically ill COVID-19 patients. We found that COVID-19 patients treated with TPE had a lower risk of mortality compared to those without TPE treatment. Since COVID-19 guidelines suggest that the use of TPE for patients with COVID-19 should be carefully implemented as the evidence of TPE efficacy was only limited to a case report, ^3^ our current findings might strengthen the evidence that the use of TPE is effective in reducing the risk of mortality among patients with COVID-19. However, in real-world implementation, special settings such as appropriate condition, target of treatment, potential complications, and particular case or comorbidity should be investigated.

Since this is the initial evidence on the potential efficacy of TPE for the management of COVID-19, several limitations should be highlighted. First, we did not include any potential confounding factors such as comorbidity, the levels of proinflammatory cytokines, and onset of disease course to describe the association between TPE and the risk of mortality rate. Second, a limited number of investigations on the use of TPE in COVID-19 management resulted in our study including only a limited number of articles. Therefore, further investigation involving a larger sample size is required. Third, the clinical setting on the use of TPE might differ among studies, and therefore, this variation might also govern the potency of bias. Fourth, among the included studies, we obtained only one randomized control trial (RCT) and three observational studies. Further meta-analyses involving only RCT studies might provide better levels of evidence.

## Conclusion

The data suggests that the use of TPE for the management of critically ill COVID-19 patients could reduce the mortality rate. The application of TPE for the management of COVID-19 should be considered in well-equipped hospitals.

## Data availability

## Underlying data

All data underlying the results are available as part of the article and no additional source data are required.

## Reporting guidelines

Figshare: PRISMA checklist for ‘the association between therapeutic plasma exchange and the risk of mortality among patients with critically ill COVID-19: a meta-analysis. https://doi.org/10.6084/m9.figshare.16622572.v1 ^16^

Data are available under the terms of the Creative Commons Attribution 4.0 International license (CC-BY 4.0).

## References

[1] Wang C Horby PW Hayden FG : A novel coronavirus outbreak of global health concern. *Lancet.* 2020;395:470–473. 10.1016/S0140-6736(20)30185-9 31986257 PMC7135038

[2] Coccia M : The impact of first and second wave of the COVID-19 pandemic in society: comparative analysis to support control measures to cope with negative effects of future infectious diseases. *Environ Res.* 2021;197:111099. 10.1016/j.envres.2021.111099 33819476 PMC8017951

[3] Rochwerg B Agarwal A Siemieniuk RA : A living WHO guideline on drugs for covid-19. *BMJ.* 2020;370:m3379. 32887691 10.1136/bmj.m3379

[4] Salluh JIF Arabi YM Binnie A : COVID-19 research in critical care: the good, the bad, and the ugly. *Intensive Care Med.* 2021;47:470–472. 10.1007/s00134-021-06367-5 33646318 PMC7917526

[5] Simon TL Snyder EL Stowell CP : *Rossi's principles of transfusion medicine.* John Wiley & Sons;2009. 10.1002/9781444303513

[6] Dundas S Murphy J Soutar RL : Effectiveness of therapeutic plasma exchange in the 1996 Lanarkshire Escherichia coli O157:H7 outbreak. *Lancet.* 1999;354:1327–1330. 10.1016/S0140-6736(99)01251-9 10533860

[7] Brocklebank V Wong EK Fielding R : Atypical haemolytic uraemic syndrome associated with a CD46 mutation triggered by Shigella flexneri. *Clin Kidney J.* 2014;7:286–288. 10.1093/ckj/sfu032 24944786 PMC4038258

[8] Burakovskii NI Sviridchuk VM : A case using plasmapheresis in treating infectious toxicosis in young infants. *Pediatriia.* 1988;98–99. 3054796

[9] Keith PD Wells AH Hodges J : The therapeutic efficacy of adjunct therapeutic plasma exchange for septic shock with multiple organ failure: a single-center experience. *Crit Care.* 2020;24:518. 10.1186/s13054-020-03241-6 32831133 PMC7443810

[10] Tabibi S Tabibi T Conic RRZ : Therapeutic Plasma Exchange: A potential Management Strategy for Critically Ill COVID-19 Patients. *J Intensive Care Med.* 2020;35:827–835. 10.1177/0885066620940259 32666875 PMC7391476

[11] Khamis F Al-Zakwani I Al Hashmi S : Therapeutic plasma exchange in adults with severe COVID-19 infection. *Int J Infect Dis.* 2020;99:214–218. 10.1016/j.ijid.2020.06.064 32585284 PMC7308750

[12] Gucyetmez B Atalan HK Sertdemir I : Therapeutic plasma exchange in patients with COVID-19 pneumonia in intensive care unit: a retrospective study. *Crit Care.* 2020;24:492. 10.1186/s13054-020-03215-8 32771054 PMC7414262

[13] Kamran SM Mirza ZE Naseem A : Therapeutic plasma exchange for coronavirus disease-2019 triggered cytokine release syndrome; a retrospective propensity matched control study. *PLoS One.* 2021;16:e0244853. 10.1371/journal.pone.0244853 33411791 PMC7790281

[14] Faqihi F Alharthy A Abdulaziz S : Therapeutic plasma exchange in patients with life-threatening COVID-19: a randomised controlled clinical trial. *Int J Antimicrob Agents.* 2021;57:106334. 10.1016/j.ijantimicag.2021.106334 33838224 PMC8024223

[15] Liberati A Altman DG Tetzlaff J : The PRISMA statement for reporting systematic reviews and meta-analyses of studies that evaluate healthcare interventions: explanation and elaboration. *BMJ.* 2009;339:b2700. 10.1136/bmj.b2700 19622552 PMC2714672

[16] Fajar J : The association between therapeutic plasma exchange and the risk of mortality among patients with critically ill COVID-19: a meta-analysis. Figshare. *Dataset.* 2021. 10.6084/m9.figshare.16622572.v1 PMC8749910 35083038

[17] Stang A : Critical evaluation of the Newcastle-Ottawa scale for the assessment of the quality of nonrandomized studies in meta-analyses. *Eur J Epidemiol.* 2010;25:603–605. 10.1007/s10654-010-9491-z 20652370

[18] Olivo SA Macedo LG Gadotti IC : Scales to assess the quality of randomized controlled trials: a systematic review. *Phys Ther.* 2008;88:156–175. 10.2522/ptj.20070147 18073267

[19] Liu J Liu S : The management of coronavirus disease 2019 (COVID-19). *J Med Virol.* 2020;92:1484–1490. 10.1002/jmv.25965 32369222 PMC7267323

[20] Downes KA Allen K Sarode R : Consanguineous hemolytic uremic syndrome secondary to *Escherichia coli* O157:H7 infection treated with aggressive therapeutic plasma exchange. *J Clin Apher.* 2001;16:155–156. 10.1002/jca.1029 11746544

[21] Reiss RF Rubinstein P Friedman-Kien A : Partial plasma exchange in patients with AIDS and Kaposi's sarcoma. Plasmapheresis in AIDS. *AIDS Res.* 1986;2:183–190. 10.1089/aid.1.1986.2.183 2428382

[22] Salim YS Faber V Skinhoj P : Plasmapheresis in the treatment of peripheral HIV neuropathy. *Ugeskr Laeger.* 1989;151:1754–1756. 2551089

[23] Bollee G Touzot M Mechai F : Plasma exchange for disseminated cryptococcosis. *Am J Kidney Dis.* 2009;53:673–676. 10.1053/j.ajkd.2008.08.019 18848378

[24] Huang C Wang Y Li X : Clinical features of patients infected with 2019 novel coronavirus in Wuhan, China. *Lancet.* 2020;395:497–506. 10.1016/S0140-6736(20)30183-5 31986264 PMC7159299

[25] Channappanavar R Perlman S : Pathogenic human coronavirus infections: causes and consequences of cytokine storm and immunopathology. *Semin Immunopathol.* 2017;39:529–539. 10.1007/s00281-017-0629-x 28466096 PMC7079893

[26] Strauss RG Ciavarella D Gilcher RO : An overview of current management. *J Clin Apher.* 1993;8:189–194. 10.1002/jca.2920080402 8113206

[27] Padmanabhan A Connelly-Smith L Aqui N : Guidelines on the Use of Therapeutic Apheresis in Clinical Practice - Evidence-Based Approach from the Writing Committee of the American Society for Apheresis: The Eighth Special Issue. *J Clin Apher.* 2019;34:171–354. 10.1002/jca.21705 31180581

[28] Bar-On YM Flamholz A Phillips R : SARS-CoV-2 (COVID-19) by the numbers. *Elife.* 2020;9. 10.7554/eLife.57309 32228860 PMC7224694

[29] Zachariah U Nair SC Goel A : Targeting raised von Willebrand factor levels and macrophage activation in severe COVID-19: Consider low volume plasma exchange and low dose steroid. *Thromb Res.* 2020;192:2. 10.1016/j.thromres.2020.05.001 32403033 PMC7198395

