# Supplementary material for: The association between therapeutic plasma exchange and the risk of mortality among patients critically ill with COVID-19: a meta-analysis
Source: F1000Res. 2021 Dec 14;10:1280. [Version 1] doi: 10.12688/f1000research.74972.1 (PMC8749910; doi:10.12688/f1000research.74972.1)
Supplement: Peer review history

# Peer review: Reviewer response for version 1

**DOI:** 10.5256/f1000research.78779.r116276

**Published:** 2022-01-13

**Authors:** Ashish Kumar

**Affiliations:** 1 Department of Critical Care Medicine, St Johns's Medical College Hospital, Bangalore, Karnataka, India

Wardhani et al., in their study titled "The association between therapeutic plasma exchange and the risk of mortality among patients critically ill with COVID-19: a meta-analysis" reported therapeutic plasma exchange to be of benefit in patients with critically ill COVID-19. I have the following comments.

 Introduction

 -None

 Methods

 - What was the statistical method used to pool data? Random/Fixed effect? What was the measure of Tau2 if IV method was used for pooling?

 Results

 - Summarize the quality of the included studies. 

 - Use GRADE criteria to grade the level of evidence.

 - Baseline Table, Table 1 needs to be elaborated more with more variables to give the reader an idea about the severity of the illness among patients included. 

 Discussion

 - The discussion needs to stress the "low quality" of included studies considering the observational nature of the study.

Are the rationale for, and objectives of, the Systematic Review clearly stated?

Yes

Is the statistical analysis and its interpretation appropriate?

Partly

Are sufficient details of the methods and analysis provided to allow replication by others?

No

Are the conclusions drawn adequately supported by the results presented in the review?

Partly

Reviewer Expertise:

Nees more methodological clarification.

I confirm that I have read this submission and believe that I have an appropriate level of expertise to confirm that it is of an acceptable scientific standard, however I have significant reservations, as outlined above.

# Peer review: Reviewer response for version 1

**DOI:** 10.5256/f1000research.78779.r116268

**Published:** 2022-01-10

**Authors:** Mahir Gachabayov

**Affiliations:** 1 Section of Colorectal Surgery, Department of Surgery, Westchester Medical Center, New York Medical College, Valhalla, NY, USA

Thank you for the opportunity of reviewing this manuscript.

 This is a systematic review and meta-analysis aiming at evaluating the use of therapeutic plasma exchange (TPE) in terms of mortality in critically ill COVID-19 patients as compared to a control group. The research question makes sense and is worth exploring as this topic has been speculated upon with anecdotal evidence. 

 The methodology of meta-analysis is adequate. The literature search was comprehensive; study screening and selection process as well as data extraction complied with PRISMA guidelines. Statistical analysis was adequate. Discussion is comprehensible and easy to read.

 Conclusion drawn was partly justified by the findings of the analysis. I would evaluate certainty of evidence generated in this meta-analysis on a GRADE scale. If certainty of evidence is assessed to be low (given the limitations), I would conclude that although TPE could reduce mortality as the findings of this study suggest, current evidence is inconclusive.

Are the rationale for, and objectives of, the Systematic Review clearly stated?

Yes

Is the statistical analysis and its interpretation appropriate?

Partly

Are sufficient details of the methods and analysis provided to allow replication by others?

Yes

Are the conclusions drawn adequately supported by the results presented in the review?

Partly

Reviewer Expertise:

clinical outcomes and evidence synthesis

I confirm that I have read this submission and believe that I have an appropriate level of expertise to confirm that it is of an acceptable scientific standard.

# Peer review: Reviewer response for version 1

**DOI:** 10.5256/f1000research.78779.r116284

**Published:** 2022-01-06

**Authors:** Guilherme Welter Wendt

**Affiliations:** 1 Psychologist and Adjunct Professor of Quantitative Research Methods, Centro de Ciências da Saúde (CCS), Universidade Estadual do Oeste do Paraná (UNIOESTE), Francisco Beltrão, Brazil

- References are needed to support these statements: "Patients critically ill with COVID-19 have been widely reported to have a poor prognosis, and theory reveals that cytokine storm might underlie this mechanism. In a cytokine storm excessive accumulation of pro-inflammatory cytokines might be responsible for the poor prognosis of COVID-19 patients".

 - "No study has found an effective treatment for the management of a cytokine storm in patients critically ill with COVID-19" - how can you prove that? Perhaps it could be informative to tone down this sentence, including "to the best of our knowledge" or provide dates and keywords used in scientific databases that resulted in zero studies.

 - These sentences could be merged, resulting in an objective, clear paragraph: "However, insufficient evidence has resulted in indecision in applying TPE for the management of critically ill COVID-19 patients. To date, TPE has been studied in Oman,11 Turkey,12 Pakistan,13 and Saudi Arabia.14 However, contradictory findings exist".

 - Under the search strategy and data extraction, please state which authors performed these tasks (as reported for the assessment methodological quality). What happened when disagreements occurred?

 - The Jada-modified scale does not appear under the section "Eligibility criteria". Should you include, like you did with the NOS?

 - In the results, you mention that studies with insufficient data were excluded. Should this appear in your inclusion/exclusion criteria too?

 - The following sentence seems incomplete: "Our analysis revealed the absence of the evidence of heterogeneity. Therefore, we applied a fixed-effect model to assess the correlation". Correlation between what?

 - The first paragraph of the discussion looks a little bit repetitive. The third paragraph also seems to repeat some of the information given at the beginning of the discussion.

 - "Previous evidence suggests that TPE might play an important role in maintaining the balance between anti and pro-inflammatory cytokines in the plasma, and might rectify the prognosis in patients with COVID-19, as reported in our study". Can you show us (cite) the evidence mentioned?

 - "our current findings might strengthen the evidence that the use of TPE is effective in reducing the risk of mortality among patients with COVID-19". Here you are not talking about severe Covid. Does it mean that TPE might benefit mild/moderate cases too?

Are the rationale for, and objectives of, the Systematic Review clearly stated?

Yes

Is the statistical analysis and its interpretation appropriate?

Yes

Are sufficient details of the methods and analysis provided to allow replication by others?

Yes

Are the conclusions drawn adequately supported by the results presented in the review?

Yes

Reviewer Expertise:

Public health, epidemiology, statistics

I confirm that I have read this submission and believe that I have an appropriate level of expertise to confirm that it is of an acceptable scientific standard, however I have significant reservations, as outlined above.

# Peer review: Reviewer response for version 1

**DOI:** 10.5256/f1000research.78779.r116258

**Published:** 2021-12-23

**Authors:** Seyi Samson Enitan

**Affiliations:** 1 Department of Medical Laboratory Science, Babcock University, Ilishan-Remo, Nigeria

This paper contributes considerably to knowledge in the field of COVID-19 management. The introduction is considered satisfactory. The authors provided background that puts the manuscript into context and allows readers outside the field to understand the purpose and significance of the study. They also identified the existing gap in knowledge that needs to be filled.  The rationale for, and objectives of, the systematic review were clearly stated.

 In this manuscript, the authors assessed the association between therapeutic plasma exchange (TPE) and the reduction of mortality of critically ill COVID-19 patients. A meta-analysis was conducted by collecting data from PubMed, Scopus, and Web of Science, in which a total of four papers were assessed. The methodology section was clearly presented to allow the reproduction of the study. Sufficient details of the analysis were also provided to allow replication by others. The statistical analysis and its interpretation were appropriate.

 The outcome of the study shows that critically ill COVID-19 patients treated with TPE had a lower risk of mortality compared to those without TPE treatment (OR: 0.21; 95% CI: 0.05, 0.85). Hence, the need for TPE advocacy with recourse to the clinical presentation of the patient.

 The study, however, is not without its attending limitations, mainly:

1. Potential confounding factors such as comorbidity, the levels of pro-inflammatory cytokines, and the onset of disease course to describe the association between TPE and the risk of mortality rate were not included.
2. The sample size was small due to the limited number of papers assessed.
3.  Bias due to variation in clinical setting in the use of TPE 
4.  Only one randomized control trial was included in the study.

The conclusion drawn was adequately supported by the data assessed in the meta-analysis.

 In all, the work is okay and the findings are worth sharing with the scientific community, however, it is important to note that the use of TPE must be done with utmost caution to forestall unexpected complications that may be associated with it.

 NB: Authors should declare whether competing interests exist or not.

Are the rationale for, and objectives of, the Systematic Review clearly stated?

Yes

Is the statistical analysis and its interpretation appropriate?

Yes

Are sufficient details of the methods and analysis provided to allow replication by others?

Yes

Are the conclusions drawn adequately supported by the results presented in the review?

Yes

Reviewer Expertise:

Immunology of infectious diseases including COVID-19, HIV/AIDS and Opportunistic infections

I confirm that I have read this submission and believe that I have an appropriate level of expertise to confirm that it is of an acceptable scientific standard.
